# Abundance and diversity of *Culicoides* Latreille (Diptera: Ceratopogonidae) in southern Ontario, Canada

**DOI:** 10.1186/s13071-023-05799-w

**Published:** 2023-06-14

**Authors:** Samantha E. Allen, Stacey L. Vigil, Tara Furukawa-Stoffer, Nicole Colucci, Aruna Ambagala, David L. Pearl, Mark G. Ruder, Claire M. Jardine, Nicole M. Nemeth

**Affiliations:** 1 Present Address: Wyoming Game and Fish Department, Veterinary Services, Laramie, USA; 2 Department of Pathobiology, Ontario Veterinary College, University of Guelph, Guelph, Canada; 3 Canadian Wildlife Health Cooperative, Ontario Veterinary College, University of Guelph, Guelph, Canada; 4 Southeastern Cooperative Wildlife Disease Study, College of Veterinary Medicine, University of Georgia, Athens, USA; 5 Canadian Food Inspection Agency, National Centre for Animal Diseases, Lethbridge, Canada; 6 Canadian Food Inspection Agency, National Centre for Foreign Animal Disease, Winnipeg, Canada; 7 Department of Population Medicine, Ontario Veterinary College, University of Guelph, Guelph, Canada; 8 Department of Pathology, University of Georgia, Athens, USA

**Keywords:** Abundance, Ceratopogonidae, *Culicoides sonorensis*, *Culicoides stellifer*, Diversity, Ontario, Canada

## Abstract

**Background:**

*Culicoides* Latreille (Diptera: Ceratopogonidae) is a genus of hematophagous midges feeding on various vertebrate hosts and serving as a vector for numerous pathogens important to livestock and wildlife health. North American pathogens include bluetongue (BT) and epizootic hemorrhagic disease (EHD) viruses. Little is known about *Culicoides* spp. distribution and abundance and species composition in Ontario, Canada, despite bordering numerous U.S. states with documented *Culicoides* spp. and BT and EHD virus activity. We sought to characterize *Culicoides* spp. distribution and abundance and to investigate whether select meteorological and ecological risk factors influenced the abundance of *Culicoides biguttatus*, *C. stellifer*, and the subgenus *Avaritia* trapped throughout southern Ontario.

**Methods:**

From June to October of 2017 to 2018, CDC-type LED light suction traps were placed on twelve livestock-associated sites across southern Ontario. *Culicoides* spp. collected were morphologically identified to the species level when possible. Associations were examined using negative binomial regression among *C. biguttatus*, *C. stellifer*, and subgenus *Avaritia* abundance, and select factors: ambient temperature, rainfall, primary livestock species, latitude, and habitat type.

**Results:**

In total, 33,905 *Culicoides* spp. midges were collected, encompassing 14 species from seven subgenera and one species group. *Culicoides sonorensis* was collected from three sites during both years. Within Ontario, the northern trapping locations had a pattern of seasonal peak abundance in August (2017) and July (2018), and the southern locations had abundance peaks in June for both years. *Culicoides biguttatus*, *C. stellifer*, and subgenus *Avaritia* were significantly more abundant if ovine was the primary livestock species at trapping sites (compared to bovine). *Culicoides stellifer* and subgenus *Avaritia* were significantly more abundant at mid- to high-temperature ranges on trap days (i.e., 17.3–20.2 and 20.3–31.0 °C compared to 9.5–17.2 °C). Additionally, subgenus *Avaritia* were significantly more abundant if rainfall 4 weeks prior was between 2.7 and 20.1 mm compared to 0.0 mm and if rainfall 8 weeks prior was between 0.1 and 2.1 mm compared to 0.0 mm.

**Conclusions:**

Results from our study describe *Culicoides* spp. distribution in southern Ontario, the potential for spread and maintenance of EHD and BT viruses, and concurrent health risks to livestock and wildlife in southern Ontario in reference to certain meteorological and ecological risk factors. We identified that *Culicoides* spp. are diverse in this province, and appear to be distinctly distributed spatially and temporally. The livestock species present, temperature, and rainfall appear to have an impact on the abundance of *C. biguttatus*, *C. stellifer*, and subgenus *Avaritia* trapped. These findings could help inform targeted surveillance, control measures, and the development of management guides for *Culicoides* spp. and EHD and BT viruses in southern Ontario, Canada.

**Graphical Abstract:**

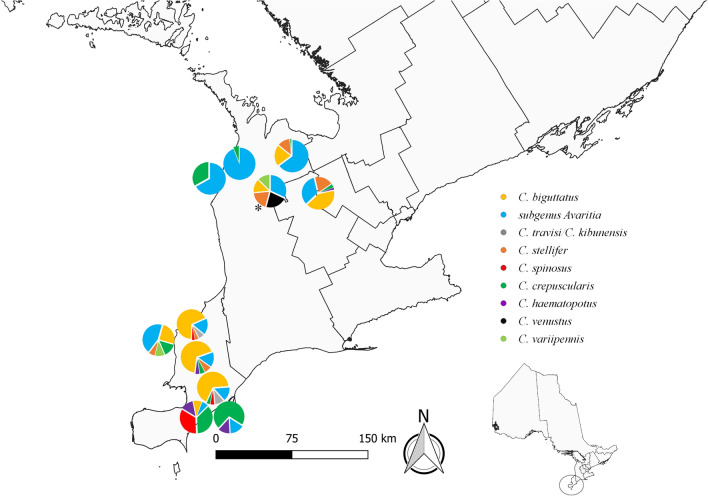

## Background

*Culicoides* Latreille (Diptera: Ceratopogonidae) is a genus of hematophagous flies, also known as biting midges, which feed on a variety of vertebrate host species [1–3]. This group includes > 1400 species worldwide that are on most major land masses across a variety of habitats, including agricultural and forested areas [1, 4, 5]. Some species of biting midges can contribute to poor performance in livestock from nuisance biting alone, and some also are competent vectors of zoonotic pathogens (e.g., Oropouche virus and *Mansonella ozzardi*) and important livestock and wildlife pathogens (e.g., Schmallenberg virus [SBV], African horse sickness virus, bluetongue virus [BTV], and epizootic hemorrhagic disease virus [EHDV]) [2, 6–8]. While in North America, two *Culicoides* spp., *C. sonorensis* and *C. insignis*, have been identified as capable of transmitting BTV and EHDV, a more complete understanding of vector competence for these viruses is lacking [9–13]. Other *Culicoides* species have been implicated as potential vectors as well (*C. stellifer*, *C. paraensis*, *C. obsoletus*, *C. haematopotus*, *C. occidentalis*, *C. venustus*) [14, 15].

These viruses pose a serious animal health threat, as BTV can cause high rates of mortality in domestic sheep [16], and both BTV and EHDV have the potential to cause high mortality among farmed and free-ranging cervids in North America, primarily white-tailed deer (*Odocoileus virginianus*) [17–19]. Recently, the frequency and geographic range of *Culicoides* spp.-driven virus outbreaks, specifically involving BTV, EHDV, and SBV, have increased in Europe, North America, and the Middle East, and have led to concerns about geographic spread [19–23]. Historically, epidemiologic patterns and the geographical distribution of both BTV and EHDV have been consistent [18]. However, the previously defined geographical limits of these viruses are changing, with outbreaks occurring more frequently and in areas not previously considered at risk [7, 19, 23, 24].

Long-standing changes in virus distribution may reflect a shifting geographic range of the vector largely in response to global climate change [19, 25]. For example, *C. sonorensis* (Wirth & Jones) has primarily been documented in portions of the western U.S. and Canada, with scattered populations east of the Mississippi River, but absent from the northeastern U.S. and eastern Canada, with *C. sonorensis* being recently recorded from a few regions of southern Ontario, Canada [7, 26–30]. While these new records are notable, *Culicoides* spp. community data are lacking from a broader region across southern Ontario and are needed to more accurately assess species diversity and abundance changes to past/current distribution. This information is crucial to determine the risk of *Culicoides* spp.-transmitted viral infections in domestic and wild ruminant populations in southern Ontario.

The emergence and re-emergence of pathogens transmitted by *Culicoides* spp. across North America, Europe, and the Middle East highlight the need for more intensive surveillance efforts that encompass vectors, viruses, and hosts. Few published studies have focused on characterizing *Culicoides* spp. composition and geographic distribution and the effects of potentially important, external factors that could contribute to species diversity and abundance [8, 19, 23]. This is especially true for areas such as Ontario, Canada, due to its northern latitude and the lack of reports of EHDV and BTV detection in livestock or wildlife prior to 2017 [23]. Therefore, the overarching goal of this study was to provide baseline data characterizing *Culicoides* spp. in sites that overlap with livestock and deer populations across southern Ontario, Canada. Specifically, our objectives were to (1) determine the relative abundance and taxonomic diversity of adult *Culicoides* spp. midges, and (2) assess the potential effects of meteorological and ecological variables on the abundance of documented *Culicoides* spp. (specifically those with highest abundance) across parts of southern Ontario, Canada.

## Methods

### Sample collection and identification

**Fig. 1:**
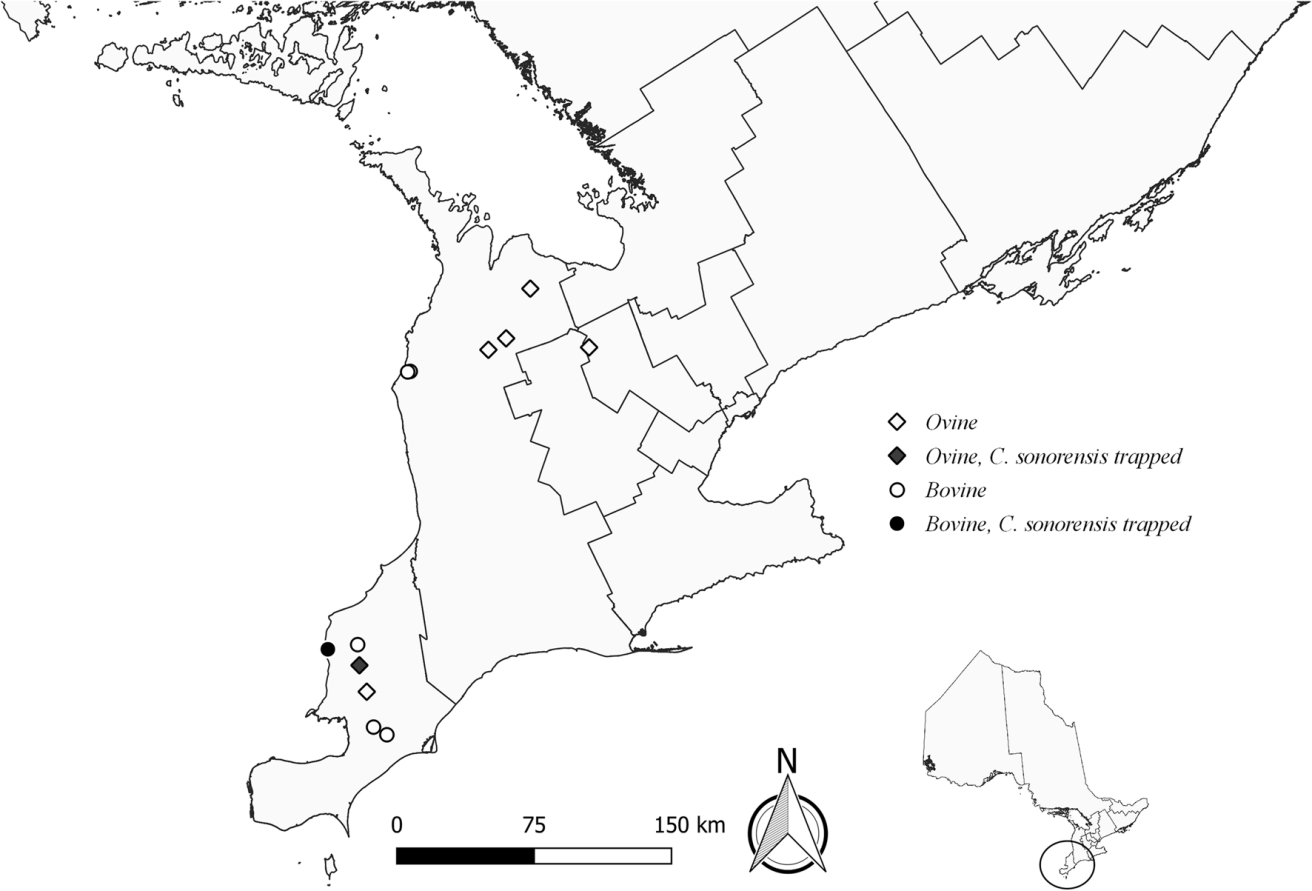
Distribution of *Culicoides* spp. trapping sites based on farm type (ovine, bovine) in southern Ontario, Canada in 2017 and 2018, including trapping sites where *Culicoides sonorensis* was reported. Lines within landmass are based on Canadian census boundaries

In 2017 and 2018, from June through October, insect surveys of southern Ontario farms were conducted and trap contents were processed as described previously [31]. Briefly, 11 farms, resulting in 12 survey sites (one farm moved between survey years), were classified as bovine (domestic cattle) or ovine (domestic sheep) primary sites (Fig. 1) and “northern” or “southern” sites. Farms consisted of primarily pastured animals. At each site, approximately every 2 weeks, two ultraviolet (UV) light-emitting diode (LED) Centers for Disease Control and Prevention (CDC) light traps (Model #2770, BioQuip Products Inc., Rancho Dominguez, CA, USA, http://www.bioquip.com) were deployed at recurring locations: within 15 m of outdoor livestock (deemed “livestock” habitat) and within 15 m of a natural area (deemed “natural” habitat, i.e., forested or wetland areas inaccessible to livestock). All collected insects were initially sorted at the University of Guelph (Guelph, Ontario), and *Culicoides* spp. were separated and shipped to the Canadian Food Inspection Agency (Lethbridge, Alberta) and the Southeastern Cooperative Wildlife Disease Study (Athens, GA) for morphological identification to species. *Culicoides* identified as members of the subgenus *Avaritia* were tallied and archived for future study.

### Peak abundance

Individual *Culicoides* spp. were sorted into groups based on their sex (male/female), species/subgenus, site, date, and trap location. The assessment of overall *Culicoides* spp., *C. biguttatus*, *C. stellifer*, and subgenus *Avaritia* peak abundance in 2017 and 2018 was standardized by use of an epidemiological week model (“epi-week”). This facilitates comparison of data across both years, as well as with datasets from other regions [32]. We defined the first epi-week of the year as the week that ended on the first Saturday in January with at least four preceding days in that month. Each epi-week began on Sunday and ended on Saturday. Therefore, in 2017, the first epi-week started on Sunday, January 1, and ended on Saturday, January 7. In 2018, the first epi-week began on Monday, January 1, and ended on Saturday, January 6.

### Statistical analyses

Statistical analyses were performed for the individual species and/or species groupings with more than 1000 individuals, as well as those with potential involvement in orbivirus transmission [7, 15, 33, 34]. These included those within the subgenus *Avaritia*, as well as *C. biguttatus* and *C. stellifer*. To investigate independent variables affecting the nightly abundance of the subgenus *Avaritia*, *C. biguttatus*, and *C. stellifer*, mixed-effects univariable and multivariable negative binomial regression models were fitted to account for overdispersion in the data [35].

Nine independent variables were included in our univariable models: primary on-site livestock species (“ovine” vs. “bovine”), habitat type (“natural” vs. “livestock”), latitude (“northern” sites vs. “southern” sites), sum of rainfall (mm; for 2-day periods that encompassed the trapping period as well as 4 and 8 weeks prior to trapping), and mean temperature (°C; for the same time periods as rainfall). Data on mean daily temperature and total rainfall were acquired from Environment Canada [36] and represented the nearest or next-nearest weather station to each site (i.e., Sarnia, ON; Strathroy-Mullifarry, ON; Chatham Kent, ON; Kingsville Ministry of the Environment, ON; Markdale, ON; Kincardine ON) for corresponding sampling time periods in 2017 and 2018.

The linearity assumption for the continuous variables of sum of rainfall (in mm, for 2-day periods that encompassed the trapping period as well as 4 and 8 weeks prior to trapping), and mean temperature (in °C; for the same time periods as rainfall) were visually assessed via lowess (i.e., locally weighted scatterplot smoothing) curves. Based on the nonlinearity of the curves, the temperature and rainfall data were each categorized into three categories based on tertiles (Table 1). In addition, the correlations between independent variables were assessed using various correlation statistics depending on the form of the variables (e.g., phi coefficients and Spearman rank correlation coefficients). If the correlation exceeded |0.8|, only one of the variables would be considered for inclusion in a multivariable model based on biological plausibility. To account for potential clustering due to repeated sampling, we initially included residence (farm site), trap ID, and trap ID date as random intercepts.

**Table 1:** Results of mixed-effects univariable negative binomial regression examining the associations between environmental factors and the abundance of *Culicoides stellifer*, *Culicoides biguttatus*, and subgenus *Avaritia* for all trapping sites in southern Ontario, Canada in 2017 and 2018

*Culicoides stellifer*	CR^a^		95% CI	*P* > z
Temperature (°C)	REF			
9.5–17.2				
17.3–20.2	10.54	2.83	39.29	< 0.001
20.3–31.0	14.54	4.21	50.26	< 0.001
Temperature4^b^ (°C)	REF			
10.3–17.3	1.03	0.41	2.55	0.957
17.4–21.0				
21.1–25.0	0.71	0.25	2.05	0.528
Temperature8^c^ (°C)	REF			
−5.0 to 16.5	1.02	0.42	2.46	0.965
16.6–20.3	0.56	0.21	1.51	0.252
20.4–25.0				
Rain (mm)	REF			
0.0–0.0	1.30	0.40	4.25	0.663
0.1–1.2				
1.3–17.9	1.87	0.84	4.12	0.123
Rain4^b^ (mm)	REF			
0.0–0.0	0.33	0.12	0.90	0.031
0.1–2.6				
2.7–20.1	0.95	0.34	2.69	0.924
Rain8^c^ (mm)	REF			
0.0–0.0	2.36	0.92	6.08	0.076
0.1–2.1				
2.2–72.0	0.72	0.28	1.83	0.485
Latitude	REF			
North				
South	0.38	0.03	4.94	0.459
Environment	REF			
Livestock	0.46	0.22	0.97	0.042
Natural				
Species	REF			
Bovine	60.65	16.28	225.97	< 0.001
Ovine				

*Culicoides biguttatus*				
Temperature (°C)	REF			
9.5–17.2	12.42	3.30	46.79	< 0.001
17.3–20.2				
20.3–31.0	4.85	1.21	19.44	0.026
Temperature4^b^ (°C)	REF			
10.3–17.3	1.56	0.31	7.93	0.593
17.4–21.0				
21.1–25.0	0.01	0.00	0.08	< 0.001
Temperature8^c^ (°C)	REF			
−5.0 to 16.5	0.02	0.01	0.08	< 0.001
16.6–20.3				
20.4–25.0	0.01	0.00	0.28	< 0.001
Rain (mm)	REF			
0.0–0.0	0.72	0.14	3.67	0.692
0.1–1.2				
1.3–17.9	0.48	0.14	1.64	0.243
Rain4^b^ (mm)	REF			
0.0–0.0	0.86	0.19	3.10	0.851
0.1–2.6				
2.7–20.1	0.10	0.02	0.49	0.005
Rain8^c^ (mm)	REF			
0.0–0.0	15.57	2.97	81.72	0.001
0.1–2.1				
2.2–72.0	0.61	0.12	3.04	0.542
Latitude	REF			
North	1.51	0.04	51.30	0.818
South				
Environment	REF			
Livestock	0.77	0.22	2.67	0.678
Natural				
Species	REF			
Bovine	140.79	13.10	1513.52	< 0.001
Ovine				

*Subgenus* *Avaritia*				
Temperature (°C)	REF			
9.5–17.2	6.03	2.90	12.53	< 0.001
17.3–20.2				
20.3–31.0	6.45	3.19	13.06	< 0.001
Temperature4^b^ (°C)	REF			
10.3–17.3	1.43	0.73	2.84	0.299
17.4–21.0				
21.1–25.0	1.46	0.70	3.01	0.311
Temperature8^c^ (°C)	REF			
−5.0 to 16.5	1.62	0.89	2.94	0.114
16.6–20.3				
20.4–25.0	0.85	0.45	1.60	0.622
Rain (mm)	REF			
0.0–0.0	0.79	0.37	1.70	0.549
0.1–1.2				
1.3–17.9	0.59	0.30	1.14	0.117
Rain 4^b^ (mm)	REF			
0.0–0.0	0.54	0.28	1.03	0.063
0.1–2.6				
2.7–20.1	1.02	0.52	1.99	0.952
Rain8^c^ (mm)	REF			
0.0–0.0	2.79	1.37	5.68	0.005
0.1–2.1				
2.2–72.0	1.11	0.55	2.23	0.770
Latitude	REF			
North	0.10	0.02	0.43	0.002
South				
Environment	REF			
Livestock	0.16	0.09	0.28	< 0.001
Natural				
Species	REF			
Bovine	12.83	3.65	45.10	< 0.001
Ovine				

*Culicoides stellifer*^a^				
Temperature (°C)	REF			
9.5–17.2	61.06	7.64	487.79	< 0.001
17.3–20.2				
20.3–31.0	130.86	14.40	1189.29	< 0.001
Temperature8^c^ (°C)	REF			
−5.0 to 16.5	0.05	0.01	0.35	0.003
16.6–20.3				
20.4–25.0	0.09	0.01	0.67	0.019
Rain4^d^ (mm)	REF			
0.0–0.0	0.03	0.01	0.16	< 0.001
0.1–2.6				
2.7–20.1	2.48	0.38	15.98	0.340
Rain8^c^ (mm)	REF			
0.0–0.0	4.88	0.94	25.25	0.059
0.1–2.1				
2.2–72.0	0.32	0.53	1.92	0.214
Species	REF			
Bovine				
Ovine	32.35	10.91	95.96	< 0.001

*Culicoides biguttatus*^e^				
Temperature4^d^ (°C)	REF			
10.3–17.3				
17.4–21.0	1.11	0.17	7.24	0.910
21.1–25.0	0.04	0.00	0.34	0.003
Temperature8^c^ (°C)	REF			
−5.0 to 16.5	0.00	0.00	0.04	< 0.001
16.6–20.3				
20.4–25.0	0.01	0.00	0.08	< 0.001
Rain4^d^ (mm)	REF			
0.0–0.0	0.07	0.01	0.43	0.004
0.1–2.6				
2.7–20.1	1.87	0.19	18.24	0.589
Rain8^c^ (mm)	REF			
0.0–0.0	2.96	0.37	23.93	0.308
0.1–2.1				
2.2–72.0	0.08	0.01	0.57	0.011
Species	REF			
Bovine				
Ovine	40.25	6.11	265.06	< 0.001

*Subgenus* *Avaritia*^f^				
Temperature (°C)	REF			
9.5–17.2	19.31	6.81	54.70	< 0.001
17.3–20.2				
20.3–31.0	33.30	9.86	112.42	< 0.001
Temperature8^c^ (°C)	REF			
−5.0 to 16.5	0.34	0.11	1.09	0.070
16.6–20.3				
20.4–25.0	0.24	0.08	0.72	0.012
Rain4^d^ (mm)	REF			
0.0–0.0	0.63	0.24	1.67	0.350
0.1–2.6				
2.7–20.1	3.18	1.16	8.73	0.025
Rain8^c^ (mm)	REF			
0.0–0.0	7.39	2.64	20.69	< 0.001
0.1–2.1				
2.2–72.0	2.11	0.74	6.03	0.162
Latitude	REF			
North	0.28	0.08	1.00	0.048
South				
Environment	REF			
Livestock	0.20	0.11	0.36	< 0.001
Natural				
Species	REF			
Bovine				
Ovine	7.60	2.11	27.35	0.002

Random effects: residence, trapID, and trapIDdate; ^a^CR, count ratio; ^b^temp4, rain4-environmental variables taken 4 weeks before trapping occurred; ^c^temp8, rain8-environmental variables taken 8 weeks before trapping occurred

Multivariable models were fitted using a manual backward elimination process. Variables were retained in the models if they were statistically significant, based on a significance level of 5% (i.e., *α* = 0.05), or acted as an explanatory antecedent or distorter variable (i.e., a confounding variable). A variable was considered a confounding variable if it was a non-intervening variable and its removal from the model resulted in a greater than 30% change in the coefficient of a statistically significant variable. Random intercepts were removed from models if their variance component was very small (i.e., less than 1 × 10^−5^) and its removal did not impact the interpretation of the fixed effects in the model. The normality and homoscedasticity of the best linear unbiased predictions (BLUPs) were assessed graphically using normal quantile plots and examining a scatter plot of the BLUPs against the predicted outcomes, respectively. In addition, we examined Pearson residuals to identify outliers.

All statistical tests were performed using STATA (STATA Intercooled 14.2; StataCorp, College Station, TX, USA).

## Results

### Descriptive statistics

**Fig. 2:**
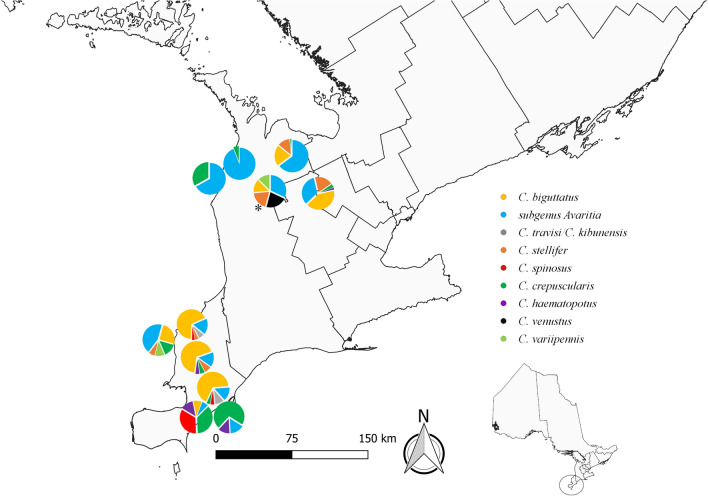
Distribution of *Culicoides* spp. in southern Ontario, Canada in 2017 and 2018. Pie charts show the percentages of adult *Culicoides* spp. trapped (including catch from each habitat type and both years). *Culicoides* spp. were included in the pie chart if ≥ 10 individuals had been trapped. This map includes the 11 farms, representing 12 sites. One site (asterisk) had a slight shift (6 km) in one location from 2017 to 2018, and was represented by one pie chart. Lines within landmass are based on Canadian census boundaries

Throughout both 2017 and 2018 (resulted in 396 trap nights of collections), a total of 33,905 individual insects identified as adult *Culicoides* spp. were trapped, encompassing 14 species belonging to seven subgenera and one species group [31] (Fig. 2).

In 2017, 19,160 individual, adult *Culicoides* spp. were trapped; these represented 14 species from seven subgenera and one species group (Table 2). Those within the subgenus *Avaritia* (Fox) were the most abundant, followed by *C. biguttatus* (Coquillett) and *C. stellifer* (Coquillett), collectively accounting for 89.9% of the 2017 collection. Female *Culicoides* spp. (97.4%; *n* = 18,667) were more abundant than males (2.6%; *n* = 493), with a female-to-male sex ratio of 38:1. *Culicoides* spp. midges were more abundant in northern (71.8%; n = 13,756) versus southern trapping sites (39.3%; *n* = 5404) (Table 3). Among *Culicoides* spp. trapped in 2017, two subgenera and 10 species were collected from both northern and southern sites (Table 3). Additionally, midges were more abundant in the traps in livestock habitat (71.9%; *n* = 13,781) than in traps in natural habitat (28.1%; *n* = 5379) (Table 4). *Culicoides sonorensis* (0.1%; *n* = 14) females were trapped from two sites in 2017 (Fig. 1).

**Table 2:** Numbers of individual, adult *Culicoides* spp. trapped across all study sites in southern Ontario, Canada in 2017 and 2018

	2017			2018		
	Female (%)	Male (%)	Total	Female (%)	Male (%)	Total
Subgenus *Avaritia* Fox	9686 (97.8)	218 (2.2)	9904	3916 (96.1)	158 (3.9)	4074
Subgenus *Beltranmyia* Vargas	0 (0.0)	0 (0.0)	0	2 (100.0)	0 (0.0)	2
* C. crepuscularis* Malloch	384 (91.0)	38 (9.0)	422	532 (82.2)	115 (17.8)	647
* C. wisconsinensis* Jones	6 (100.0)	0 (0.0)	6	2 (100.0)	0 (0.0)	2
Subgenus *Diphaomyia* Vargas	0 (0.0)	0 (0.0)	0	0 (0.0)	0 (0.0)	0
* C. baueri* Hoffman	12 (100.0)	0 (0.0)	12	5 (100.0)	0 (0.0)	5
* C. bergi* Cochrane	1 (100.0)	0 (0.0)	1	1 (33.3)	2 (66.7)	3
* C. haematopotus* Malloch	204 (87.9)	28 (12.1)	232	313 (90.5)	33 (9.5)	346
Subgenus *Hoffmania* Fox	0 (0.0)	0 (0.0)	0	0 (0.0)	0 (0.0)	0
* C. venustus* Hoffman	141 (87.0)	21 (13.0)	162	98 (97.0)	3 (3.0)	101
Subgenus *Monoculicoides* Khalaf	48 (50.0)	48 (50.0)	96	7 (43.8)	9 (56.2)	16
* C. sonorensis* Wirth and Jones	14 (100.0)	0 (0.0)	14	2 (100.0)	0 (0.0)	2
* C. variipennis* Coquillett	187 (100.0)	0 (0.0)	187	67 (100.0)	0 (0.0)	67
Subgenus *Oecacta* Poey	0 (0.0)	0 (0.0)	0	0 (0.0)	0 (0.0)	0
* C. stellifer* Coquillett	2226 (97.8)	50 (2.2)	2276	1274 (98.0)	26 (2.0)	1300
Subgenus *Silvaticulicoides* Glukhova	0 (0.0)	0 (0.0)	0	0 (0.0)	0 (0.0)	0
* C. biguttatus* Coquillett	5040 (100.0)	0 (0.0)	5040	7319 (100.0)	0 (0.0)	7319
* C. spinosus* Root and Hoffman	252 (99.6)	1 (0.4)	253	302 (100.0)	0 (0.0)	302
Subgenus unplaced, *piliferus* species group	36 (97.3)	1 (2.7)	37	77 (97.5)	2 (2.5)	79
* C. bickleyi* Wirth and Hubert	7 (100.0)	0 (0.0)	7	1 (100.0)	0 (0.0)	1
* C. denticulatus* Wirth and Hubert	4 (100.0)	0 (0.0)	4	1 (100.0)	0 (0.0)	1
* C. downesi* Wirth and Hubert	1 (100.0)	0 (0.0)	1	11 (100.0)	0 (0.0)	11
Miscellaneous unplaced species	0 (0.0)	0 (0.0)	0	0 (0.0)	0 (0.0)	0
* C. travisi/C. kibunensis* group	360 (99.7)	1 (0.3)	361	346 (100.0)	0 (0.0)	346
Unknown *Culicoides* spp.	58 (40.0)	87 (60.0)	145	46 (38.0)	75 (62.0)	121
Total	18667 (97.4)	493 (2.6)	19160	14322 (97.1)	423 (2.9)	14745

^a^Random effect: trapIDdate; ^b^CR-Count Ratio; ^c^temp8, rain8-environmental variables taken eight weeks before trapping occurred temp4; ^d^rain4-environmental variables taken four weeks before trapping occurred; ^e^Random effect: residence; ^f^Random effects: residence and trapIDdate

**Table 3:** Numbers of individual, adult *Culicoides* spp. trapped in northern and southern site locations in southern Ontario, Canada in 2017 and 2018

	2017			2018		
	Northern (%)	Southern (%)	Total	Northern (%)	Southern (%)	Total
Subgenus *Avaritia* Fox	8487 (85.7)	1417 (14.3)	9904	3662 (89.9)	412 (10.1)	4074
Subgenus *Beltranmyia* Vargas	0 (0.0)	0 (0.0)	0	1 (50.0)	1 (50.0)	2
* C. crepuscularis* Malloch	180 (42.7)	242 (57.3)	422	273 (42.2)	374 (57.8)	647
* C. wisconsinensis* Jones	2 (33.3)	4 (66.7)	6	0 (0.0)	2 (100.0)	2
Subgenus *Diphaomyia* Vargas	0 (0.0)	0 (0.0)	0	0 (0.0)	0 (0.0)	0
* C. baueri* Hoffman	12 (100.0)	0 (0.0)	12	5 (100.0)	0 (0.0)	5
* C. bergi* Cochrane	1 (100.0)	0 (0.0)	1	3 (100.0)	0 (0.0)	3
* C. haematopotus* Malloch	75 (32.3)	157 (67.7)	232	90 (26.0)	256 (74.0)	346
Subgenus *Hoffmania* Fox	0 (0.0)	0 (0.0)	0	0 (0.0)	0 (0.0)	0
* C. venustus* Hoffman	157 (96.9)	5 (3.1)	162	100 (99.0)	1 (1.0)	101
Subgenus *Monoculicoides* Khalaf	31 (32.3)	65 (67.7)	96	5 (31.2)	11 (68.8)	16
* C. sonorensis* Wirth and Jones	0 (0.0)	14 (100.0)	14	0 (0.0)	2 (100.0)	2
* C. variipennis* Coquillett	101 (54.0)	86 (46.0)	187	38 (56.7)	29 (43.3)	67
Subgenus *Oecacta* Poey	0 (0.0)	0 (0.0)	0	0 (0.0)	0 (0.0)	0
* C. stellifer* Coquillett	1872 (82.2)	404 (17.8)	2276	1095 (84.2)	205 (15.8)	1300
Subgenus *Silvaticulicoides* Glukhova	0 (0.0)	0 (0.0)	0	0 (0.0)	0 (0.0)	0
* C. biguttatus* Coquillett	2609 (51.8)	2431 (48.2)	5040	2655 (36.3)	4664 (63.7)	7319
* C. spinosus* Root and Hoffman	67 (26.5)	186 (73.5)	253	81 (26.8)	221 (73.2)	302
Subgenus unplaced, *piliferus* species group	37 (100.0)	0 (0.0)	37	79 (100.0)	0 (0.0)	79
* C. bickleyi* Wirth and Hubert	7 (100.0)	0 (0.0)	7	1 (100.0)	0 (0.0)	1
* C. denticulatus* Wirth and Hubert	4 (100.0)	0 (0.0)	4	1 (100.0)	0 (0.0)	1
* C. downesi* Wirth and Hubert	1 (100.0)	0 (0.0)	1	11 (100.0)	0 (0.0)	11
Miscellaneous unplaced species	0 (0.0)	0 (0.0)	0	0 (0.0)	0 (0.0)	0
* C. travisi/C. kibunensis* group	49 (13.6)	312 (86.4)	361	6 (1.7)	340 (98.3)	346
Unknown *Culicoides* spp.	64 (44.1)	81 (55.9)	145	46 (38.0)	75 (62.0)	121
Total	13756 (71.8)	5404 (28.2)	19160	8152 (55.3)	6593 (44.7)	14745

**Table 4:** Numbers of individual, adult *Culicoides* spp. trapped in livestock and natural habitats sites in southern Ontario, Canada in 2017 and 2018

	2017			2018		
	Livestock (%)	Natural (%)	Total	Livestock (%)	Natural (%)	Total
Subgenus *Avaritia* Fox	9276 (93.7)	628 (6.3)	9904	2824 (69.3)	1250 (30.7)	4074
Subgenus *Beltranmyia* Vargas	0 (0.0)	0 (0.0)	0	0 (0.0)	2 (100.0)	2
* C. crepuscularis* Malloch	251 (59.5)	171 (40.5)	422	260 (40.2)	387 (59.8)	647
* C. wisconsinensis* Jones	5 (83.3)	1 (16.7)	6	1 (50.0)	1 (50.0)	2
Subgenus *Diphaomyia* Vargas	0 (0.0)	0 (0.0)	0	0 (0.0)	0 (0.0)	0
* C. baueri* Hoffman	8 (66.7)	4 (33.3)	12	1 (20.0)	4 (80.0)	5
* C. bergi* Cochrane	0 (0.0)	1 (100.0)	1	0 (0.0)	3 (100.0)	3
* C. haematopotus* Malloch	46 (19.8)	186 (80.2)	232	49 (14.2)	297 (85.8)	346
Subgenus *Hoffmania* Fox	0 (0.0)	0 (0.0)	0	0 (0.0)	0 (0.0)	0
* C. venustus* Hoffman	118 (72.8)	44 (27.2)	162	53 (52.5)	48 (47.5)	101
Subgenus *Monoculicoides* Khalaf	86 (89.6)	10 (10.4)	96	13 (81.2)	3 (18.8)	16
* C. sonorensis* Wirth and Jones	12 (85.7)	2 (14.3)	14	2 (100.0)	0 (0.0)	2
* C. variipennis* Coquillett	158 (84.5)	29 (15.5)	187	40 (59.7)	27 (40.3)	67
Subgenus *Oecacta* Poey	0 (0.0)	0 (0.0)	0	0 (0.0)	0 (0.0)	0
* C. stellifer* Coquillett	1846 (81.1)	430 (18.9)	2276	364 (28.0)	936 (72.0)	1300
Subgenus *Silvaticulicoides* Glukhova	0 (0.0)	0 (0.0)	0	0 (0.0)	0 (0.0)	0
* C. biguttatus* Coquillett	1809 (35.9)	3231 (64.1)	5040	5104 (69.7)	2215 (30.3)	7319
* C. spinosus* Root and Hoffman	89 (35.2)	164 (64.8)	253	149 (49.3)	153 (50.7)	302
Subgenus unplaced, *piliferus* species group	12 (32.4)	25 (67.6)	37	11 (13.9)	68 (86.1)	79
* C. bickleyi* Wirth and Hubert	0 (0.0)	7 (100.0)	7	0 (0.0)	1 (100.0)	1
* C. denticulatus* Wirth and Hubert	0 (0.0)	4 (100.0)	4	0 (0.0)	1 (100.0)	1
* C. downesi* Wirth and Hubert	0 (0.0)	1 (100.0)	1	1 (9.1)	10 (90.9)	11
Miscellaneous unplaced species	0 (0.0)	0 (0.0)	0	0 (0.0)	0 (0.0)	0
* C. travisi/C. kibunensis* group	24 (6.6)	337 (93.4)	361	8 (2.3)	338 (97.7)	346
Unknown *Culicoides* spp.	41 (28.3)	104 (71.7)	145	24 (19.8)	97 (80.2)	121
Total	13781 (71.9)	5379 (28.1)	19160	8904 (60.4)	5841 (39.6)	14745

^a^ Random effect: trapIDdate; ^b^CR-Count Ratio; ^c^temp8, rain8-environmental variables taken eight weeks before trapping occurred temp4, ^d^rain4-environmental variables taken four weeks before trapping occurred; ^e^Random effect: residence; ^f^Random effects: residence and trapIDdate.

In 2018, we trapped a total of 14,745 individual, adult *Culicoides* spp. that represented 14 species from seven subgenera and one species group (Table 2). *Culicoides biguttatus* (Coquillett) was the most abundant species, followed by species within the subgenus *Avaritia* (Fox), and *C. stellifer* (Coquillett); these species accounted for 86.1% of the 2018 collection. Female *Culicoides* spp. (97.1%; *n* = 14,322) were more abundant than males (2.9%; *n* = 423), with a female-to-male sex ratio of 34:1. *Culicoides* spp. were more abundant in northern (55.3%; *n* = 8152) versus southern sites (44.7%; *n* = 6593) (Table 3). Three subgenera and eight species were collected from both northern and southern sites (Table 3). Additionally, *Culicoides* spp. were more abundant in livestock habitat (60.4%; *n* = 8904) than natural habitats (39.6%; *n* = 5841) (Table 4). *Culicoides sonorensis* (0.01%; *n* = 2) females were trapped at one site in 2018. Morphological identification to species of 270 specimens was precluded by post-collection artifacts and these were classified as *Culicoides* spp.

**Fig. 3:**
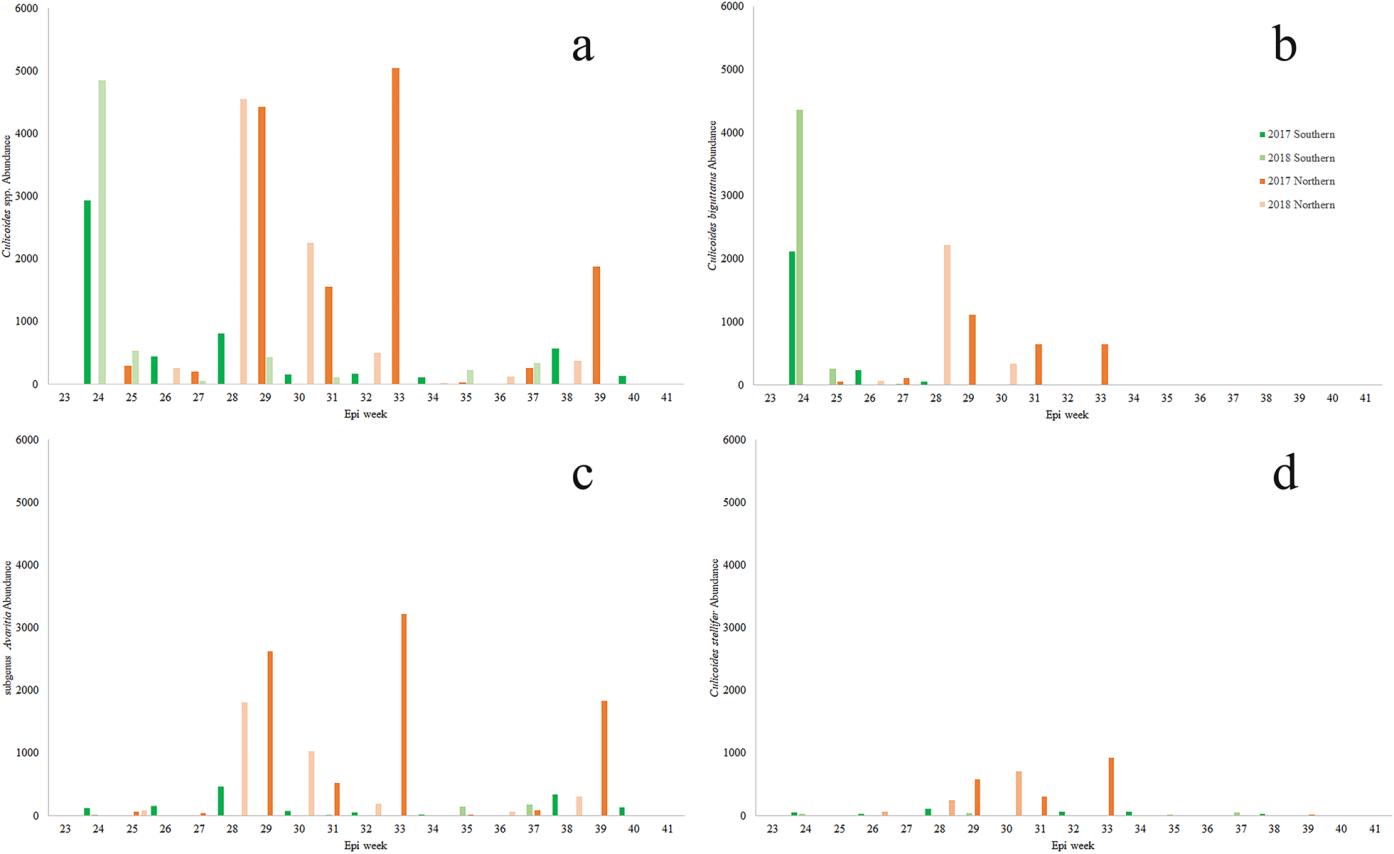
Abundance of adult *Culicoides* spp. (all) (**a**), *Culicoides biguttatus* (**b**), subgenus *Avaritia* (**c**) and *C. stellifer* (**d**) by epi-week (Central Massachusetts Mosquito Control Project 2022) from northern and southern locations in southern Ontario, Canada (June–October 2017 and 2018). For southern locations in 2017, trapping occurred in epi-week 24, 26, 28, 29, 30, 31, 32, 34, 36, 38, 40; and for 2018: 24, 25, 27, 29, 31, 34, 35, 37, 41. For northern locations in 2017, trapping occurred in epi-week 25, 27, 29, 31, 33, 35, 37, 39, 41; and for 2018: 23, 26, 28, 30, 32, 34, 36, 38, 40

In 2017, adult *Culicoides* spp. peak abundance across the northern locations had multiple crests, including mid-July, mid-August, and late September [July 16–22 (epi-week 29); *C. biguttatus*, *C. stellifer*, and subgenus *Avaritia*), August 13–16 (epi-week 33); *C. stellifer* and subgenus *Avaritia*), and September 24–30 (epi-week 39); subgenus *Avaritia*] (Fig. 3). Across southern locations, there was a peak of abundance during mid-June [June 11–17 (epi-week 24); *C. biguttatus*]. In 2018, adult *Culicoides* spp. peak abundance in the northern locations had two activity peaks in mid-July and the end of July [July 8–14 (epi-week 28); *C. biguttatus* and subgenus *Avaritia* and July 22–28 (epi-week 30); *C. stellifer*]. For the southern locations, a peak of abundance occurred in mid-June [June 10–16 (epi-week 24); *C. biguttatus*] (Fig. 3).

### Univariable mixed models

Overall, the temperature throughout the two study years ranged from 9.5 to 31.0 °C on trap days, 10.3–25.0 °C 4 weeks prior, and −5 to 25.0 °C 8 weeks prior (Table 1). Rainfall throughout the study ranged from 0.0 to 17.9 mm on trap days, 0.0–20.1 mm 4 weeks prior, and 0.0–72.0 mm 8 weeks prior (Table 1). Based on univariable analyses, *C. stellifer* was significantly more abundant at mid- to high-temperature ranges on trap days (i.e., 17.3–20.2 and 20.3–31.0 °C compared to 9.5–17.2 °C) and on sites with ovine as the primary livestock type compared to bovine (Table 1). *Culicoides stellifer* was significantly less abundant if the rainfall 4 weeks prior was between 0.1 and 2.6 mm compared to 0.0 mm and if insects were trapped in natural habitats compared to livestock habitats (Table 1). *Culicoides biguttatus* was significantly more abundant if temperatures on trap days were in mid to high ranges (i.e., 17.3–20.2 and 20.3–31.0 °C compared to 9.5–17.2 °C), if the rainfall 8 weeks prior was from 0.1 to 2.1 mm (compared to 0.0 mm) and with ovine as the primary livestock type (compared to bovine) (Table 1). *Culicoides biguttatus* was significantly less abundant if temperatures 4 weeks prior were higher (i.e., 21.1–25.0 °C compared to 10.3–17.3 °C), 8 weeks prior when 16.6–20.3 and 20.4–25.0 °C compared to −5.0 to 16.5 °C, and if rainfall 4 weeks prior was from 2.7 to 20.1 mm (compared to 0.0 mm) (Table 1). Subgenus *Avaritia* was significantly more abundant if the temperature on trap days was in mid- to high-temperature ranges (i.e., 17.3–20.2 and 20.3–31.0 °C compared to 9.5–17.2 °C), if rainfall 8 weeks prior was between 0.1 and 2.1 mm compared to 0.0 mm and at sites with ovine as the primary livestock type compared to bovine (Table 1). Subgenus *Avaritia* were significantly less abundant at natural habitats compared to livestock habitats and at southern sites compared to northern sites (Table 1).

### Multivariable mixed models

Based on multivariable analysis, *C. stellifer* abundance was significantly greater on farms with ovine livestock compared to bovine, and with temperatures on trap days at 17.3–20.2 °C or 20.3–31.0 °C compared to 9.5–17.2 °C. *Culicoides stellifer* abundance was significantly lower if rainfall 4 weeks prior to trapping was between 0.1 and 2.6 mm compared to 0.0 mm and the temperature 8 weeks prior was 16.6–20.3 or 20.4–25.0 °C compared to −5.0 to 16.5 °C (Table 1).

The abundance of *Culicoides biguttatus* was significantly greater on farms with ovine livestock compared to bovine, and were significantly lower when temperature 4 weeks prior was 21.1–25.0 °C compared to 10.3–17.3 °C, rainfall 4 weeks prior was 0.1–2.6 mm compared to 0.0 mm, temperature 8 weeks prior was 16.6–20.3 °C or 20.4–25.0 °C compared to −5.0 to 16.5 °C, and rainfall 8 weeks prior was 2.2–72.0 mm compared to 0.0 mm (Table 1).

The abundance of subgenus *Avaritia* was significantly greater on farms with ovine livestock compared to bovine, with temperature on trap days at 17.3–20.2 °C or 20.3–31.0 °C compared to 9.5–17.2 °C, rainfall 4 weeks prior at 2.7–20.1 mm compared to 0.0 mm, and rainfall 8 weeks prior at 0.1–2.1 mm compared to 0.0 mm. The abundance of subgenus *Avaritia* was significantly lower with temperature 8 weeks prior at 20.4–25.0 °C compared to −5.0 to 16.5 °C, in natural habitat compared to livestock, and in farms located further south (Table 1).

The BLUPs for all models met the assumptions of normality and homoscedasticity. Potential outliers were identified, but their removal from the models did not change the interpretation of the models presented and no recording errors were identified.

## Discussion

Although *Culicoides* spp. can be a severe nuisance to humans and animals, they pose a more substantial threat as biological vectors of viral pathogens [1]. Orbiviruses (e.g., BTV and EHDV) are transmitted by *Culicoides* spp. and threaten wildlife and livestock, especially naïve populations in northern latitudes, such as Ontario, Canada [23]. The health risk to these populations is even greater based on the recent orbivirus incursion into northern latitudes across several continents [18, 23, 37]. Based on these ongoing northern incursions, which have been well documented in the United States [19], we conducted a comprehensive survey of *Culicoides* spp. from sites throughout southern Ontario, Canada over two field seasons. We observed that within southern Ontario, more northern *Culicoides* spp. trapping locations had a pattern of seasonal peak abundance in August (2017) and July (2018), and southern locations had abundance peaks in mid-June for both years. Overall, a higher richness of *Culicoides* spp. (including two species and one species group) were collected in light traps at sites where ovine was the primary livestock type. A known BTV and EHDV vector (*C. sonorensis*) was among the *Culicoides* species identified, as well as potential vectors, *C. stellifer* and *C. venustus*.

Identifying the seasonal peak abundance (i.e., generation emergence) of targeted vector species of public, livestock, or wildlife health importance can assist in the development of risk management and future surveillance approaches. It can also help identify mitigation strategies, such as adjusting the timing of livestock management activities (e.g., shearing sheep, pasture rotation, moving animals indoors) to minimize skin contact and thus biting [38]. *Culicoides* spp. data, such as seasonal peak abundance, for Ontario are scarce, and the landscapes and latitudes are highly varied, making it difficult to compare results across studies and regions. For the northern sites in our study, we identified numerous peaks but the overall seasonal abundance peak was in mid- to late summer of both years (i.e., August 2017 and July 2018). Our findings for northern locations differ from the seasonal peaks previously identified in Ontario [39], but resemble those in Northern Ireland and southeast England where some species (e.g., *C. obsoletus*, subgenus *Avaritia*) have two to three distinct abundance peaks [40, 41] usually in late July and early August [41]. In our study, seasonal abundance peaked in mid-June in more southern Ontario locations for both years of study, with similar observed abundance peaks as previously described in more eastern Ontario locations. Specifically, Jewiss-Gaines [39] reported numerous sequential, annual (2013–2017), seasonal *Culicoides* spp. abundance peaks in June in St. Catharines, Ontario. This site, located close to the border with the U.S. (at Niagara Falls, New York), is approximately 300 km northeast of our nearest southern location.

*Culicoides* spp. reproduction and survivability in any given region are influenced in part by landscape and climatic variables [1, 42–44]. Habitat preferences, including host species availability and larval habitat, will impact the ability and frequency of *Culicoides* reproduction, which in turn will dictate the abundance and regional diversity of *Culicoides* spp. in a given area and year [41, 45, 46]. We observed higher abundance of some *Culicoides* spp. at sites where sheep (ovine) were the primary livestock type. While some *Culicoides* spp. females have shown host species preferences [6, 47], preferences for ovine-occupied habitats have not yet been shown for *C. biguttatus*, *C. stellifer*, or subgenus *Avaritia* within North America. In general, these species are considered mammal-biting generalist feeders, including white-tailed deer in some regions, utilizing a variety of avian and mammalian hosts [6, 46, 48–50]. Feeding selection in some cases may be attributed to spatial overlap of vectors and hosts, not the host preference itself [46]. Additionally, site management could be inadvertently increasing the success of *Culicoides* larval stages. Our observation of increased midge abundance at sites where ovine were the primary livestock type could be due to differences in Ontario livestock management systems between cattle and sheep (e.g., water and waste management systems), how different hosts use the landscape (e.g., their comfort with and thereby proximity to traps), associated landscape differences (e.g., water systems, common ground substrates), or some unrecognized factor(s) (e.g., insecticide/antiparasitic use) unrelated to host type [41, 51].

In addition to landscape, climatic conditions may affect *Culicoides* spp. abundance [1, 42–44]. For example, in our study, temperature appeared to impact the abundance of *C. stellifer*, subgenus *Avaritia*, and *C. biguttatus*. While temperature has been proposed to positively influence *Culicoides* spp. abundance in some temperate regions [52, 53], there are temperature thresholds at which abundance is negatively impacted for some *Culicoides* spp. [5]. In our study, daily temperatures did not exceed 31.0 °C so we were not able to establish temperature thresholds for the *Culicoides* spp. detected. For example, *C. biguttatus* numbers decreased with increasing seasonal temperatures in Georgia and eastern Tennessee, USA [48, 54], indicating that this vernal species is not tolerant of higher temperatures, and its survival or activity may be negatively impacted by higher temperatures. This could explain why temperature categories from 4 and 8 weeks prior to insect collection appeared to correlate to decreased abundance of *C. biguttatus* in Ontario in our study.

Additional climatic factors also are important, as higher rainfall amounts can impact breeding and thus *Culicoides* abundance by lowering the temperature and raising humidity [53, 55]. In our study, rainfall amounts 4 and 8 weeks prior to trapping significantly impacted the abundance of *C. biguttatus* and was associated with both a decrease in abundance [4 weeks prior (0.1–2.6 mm category), and 8 weeks prior (2.2–72.0 mm category)]. Too much rain may discourage midges from activities such as foraging or mate-seeking [53], which may explain the decrease in abundance of *C. biguttatus* in the higher rain categories. *Culicoides biguttatus* tend to emerge early in the season (i.e., spring) and emergence longevity depends on environmental moisture levels [54, 56]. In our study, an increase in rainfall amounts 4 and 8 weeks prior to trapping may have adversely affected *C. biguttatus* abundance by disrupting breeding sites and inhibiting feeding and mating [53, 54]. In terms of increasing abundance with a moderate increase in rainfall (such as in our study with subgenus *Avaritia*), *Culicoides* species do require water/moisture in many cases for development and survival [1, 2, 5, 7]. Our data suggest that the effects of environmental factors such as temperature and precipitation vary by species and species group, and correspond to species-specific phenological and environmental constraints. Additionally, some environmental variables may indirectly impact others, further increasing the complexity of vector–host–virus interactions within the environment. Such additional interactions in these systems are not accounted for in the present analysis but are an important consideration in devising region-specific, vector control strategies aimed at mitigation of virus transmission (such as eliminating/reducing larval development sites).

Despite their importance as vectors of EHDV and BTV, the geographic distribution and abundance of *Culicoides* spp., as well as species-specific vectorial capacity, are poorly understood [15, 18]. In North America, only two *Culicoides* spp. have been confirmed as vectors of BTV and EHDV (*C. sonorensis* and *C. insignis*) [10, 12, 13]. In Ontario, we identified a small number of *C. sonorensis*, mainly in southern sites close to livestock. Livestock proximity was not surprising, since *C. sonorensis* larvae prefer “waste-enhanced mud” (i.e., manure-polluted water) [7, 57, 58]. We identified additional species that may be competent vectors and facilitate EHDV and BTV spread in the region (e.g., *C. stellifer*, *C. spinosus*, and *C. venustus*) [11, 15, 19]. *Culicoides stellifer* inhabits temperate regions throughout most of the United States (with the exception of the Pacific Northwest) and eastern Canada, from Ontario to Nova Scotia [49, 59]. While *C. stellifer* was recorded throughout our trapping sites, numbers were lower at southern sites. *Culicoides spinosus* has been found in Alberta eastward to Nova Scotia and south to Nebraska, Louisiana, and Florida [59]. In our study, *C. spinosus* was found throughout the study range but was lower in number at northern sites. *Culicoides venustus* has been documented in Maryland, south to Nebraska, Louisiana, and Florida, and in Ontario eastward to Nova Scotia [59]. We recovered them at multiple study sites in southern Ontario, mainly at more northern locations. Due to their recognized importance to agriculthealth in the U.S., additional research on these *Culicoides* species is needed [19].

The distribution of *Culicoides* spp. as well as other arthropod vectors is changing, and in some cases expanding, due to altered landscape and climate dynamics [19, 28, 29, 60]. In particular, *C. sonorensis* and *C. insignis* may be undergoing a northward expansion in North America [28, 29]. Over 1400 *Culicoides* spp. have been documented worldwide, and while characterization of taxonomic diversity, composition, and distribution of many species is ongoing, such baseline information is lacking in many northern latitudes. We identified *C. sonorensis* at three of our southern Ontario study sites. In Canada, *C. sonorensis* was previously believed to exist only in western regions, mainly British Columbia and Alberta [26–28]. However, *C. sonorensis* recently was identified in the public health regions of Lambton, Oxford, Hamilton, and Niagara across the southwestern portion of southern Ontario [28]. While we have only confirmed a small number of individuals, our study reinforces the previous findings by identifying *C. sonorensis* from additional sites in the southwestern portion of southern Ontario (Lambton/Sarnia Fig. 1). Our results suggest that *C. sonorensis* may be more widespread in the southwestern region of Ontario than previously known. *Culicoides sonorensis* may have already been present in this area but was not previously identified. Historical surveillance in Ontario has been minimal. Our continued identification of this vector suggests that the province requires continued vigilance and expanded surveillance because this area could be at a higher risk for BTV/EHDV incursion and establishment. There were individuals within subgenus *Monoculicoides* that could not be further classified in our study (Tables 2, 3, 4). With the advancements in genetic differentiation [30], this could assist future work where hybridization and cryptic species are present/possible.

Our study has limitations common to previous insect-based surveillance studies, including biases associated with trapping (e.g., frequency of trapping, trap light, trap height, attractant used), sampling sites (e.g., habitat type and microhabitats, and proximity to other habitats), and challenges in taxonomic identification. While suction traps are the gold standard for insect surveillance studies, they are inherently biased. Species diversity and composition in a given trap vary based on site selection, trap type (e.g., CDC, OVI [ovitrap], Rothamsted), attractant use (e.g., light [LED/UV], carbon dioxide), and placement (i.e., height) [46, 61–65], thus affecting our understanding of spatial–temporal dynamics. In addition, specific trap types often target a single vector life stage (e.g., larva vs. adult), which may limit understanding of the implications of vector presence in a given region. Our site selections were opportunistic based on voluntary farmer participation and available farm types in our target locations, which created nonuniform coverage of the landscape. The present study targeted adult *Culicoides* spp. in flight, both through trap type selection and habitat/trap placement, whereas inclusion of larval trapping and resting sites (e.g., tree cover) would have provided a more holistic picture of *Culicoides* vector biology in the study region. In addition, identifying *Culicoides* spp. by morphological structural traits requires extensive training and specialized expertise, and occasionally, molecular confirmation. This was the case for the close relatives in subgenus *Monoculicoides*, *C. sonorensis*, and *C. variipennis* and for the subgenus *Avaritia* which can comprise a number of species. In some cases, we could not confirm the species, which may have resulted in artificially low numbers of *C. sonorensis*. For subgenus *Avaritia*, which includes species that are morphologically similar and often co-occur, accurate species identification generally requires specimen dissection and slide mounting to closely examine mouth parts. This process would have not been feasible considering the large number of specimens collected. Additionally, as we grouped our seasonal abundance data for an overall picture of what occurred over trapping seasons, expanding upon this work would provide a more detailed picture of these different species.

## Conclusions

We identified and quantified numerous *Culicoides* species, subgenera, and species groups from different sites across southern Ontario, Canada, and identified environmental variables that could impact regional vector abundance. The presence of *Culicoides* spp. in the study region overlaps with habitats and landscapes that are home to both domestic and wild animals at risk of infection and disease due to *Culicoides* spp.-transmitted pathogens, such as EHDV and BTV. In our study, we identified that *Culicoides* spp. appear to be distinctly spatially and temporally distributed. The livestock species present, temperature, and rainfall appear to have an impact on the abundance of *Culicoides biguttatus*, *C. stellifer*, and subgenus *Avaritia* trapped. A more complete understanding of the diversity and abundance of this important arthropod group, as well as aspects of their biology and the surrounding environment, requires additional work. Future studies in southern Ontario should focus on other *Culicoides* species of concern (e.g., *C. stellifer*) and include multiple consecutive (more than 2 years) with year-round sampling seasons. The resulting data would expand upon and improve our understanding of the present study results and assist in the development of risk assessments and mitigation tactics.

## Data Availability

All data generated and analyzed during this study are included in this published article.

## Abbreviations

BTV: Bluetongue virus
CDC: Centers for Disease Control and Prevention
EHDV: Epizootic hemorrhagic disease virus
CR: Count ratio
LED: Light-emitting diode
UV: Ultraviolet
